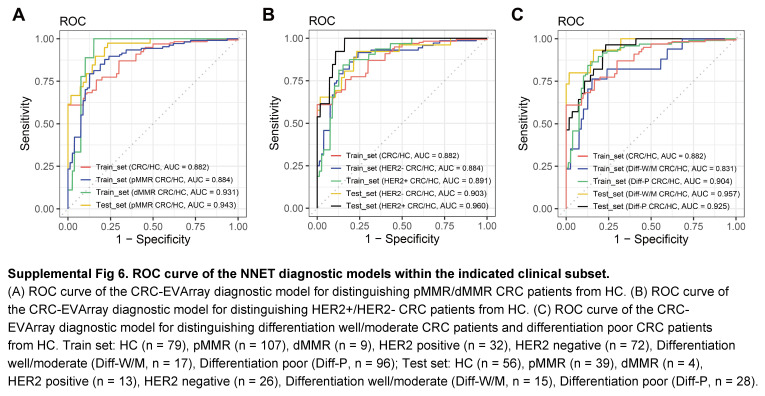# Identification and validation of the surface proteins FIBG, PDGF-β, and TGF-β on serum extracellular vesicles for non-invasive detection of colorectal cancer: experimental study

**DOI:** 10.1097/JS9.0000000000001533

**Published:** 2024-05-03

**Authors:** Zhijian Huang, Cuncan Deng, Caiqi Ma, Guirong He, Jian Tao, Lijun Zhang, Xiaoyun Hu, Yanfang Mo, Lumei Qiu, Ningfang Zhang, Chuanghua Luo, Shan Xing, Jinye Xie, Haofan Yin

**Affiliations:** a Department of Pathology, The Seventh Affiliated Hospital of Sun Yat-Sen University; b Digestive Diseases Center, The Seventh Affiliated Hospital of Sun Yat-sen University; c Department of Laboratory Medicine, Shenzhen People’s Hospital (The Second Clinical Medical College, Jinan University; The First Affiliated Hospital, Southern University of Science and Technology), Shenzhen; d Department of Laboratory Medicine, Zhongshan City People’s Hospital, Zhongshan; e Department of Oncology, Key Laboratory of Biological Targeting Diagnosis, Therapy and Rehabilitation of Guangdong Higher Education Institutes, The Fifth Affiliated Hospital of Guangzhou Medical University; f Department of Clinical Laboratory, State Key Laboratory of Oncology in South China, Sun Yat-sen University Cancer Center; g State Key Laboratory of Oncology in South China, Collaborative Innovation Center for Cancer Medicine, Sun Yat-sen University Cancer Center, Guangzhou, Guangdong, China

**Keywords:** Biomarker, colorectal cancer, extracellular vesicles, machine-learning

## Abstract

**Objectives:**

The absence of non-invasive biomarkers for the early diagnosis of colorectal cancer (CRC) has contributed to poor prognosis. Extracellular vesicles (EVs) have emerged as promising candidates for cancer monitoring using liquid biopsy. However, the complexity of EVs isolation procedures and the absence of clear targets for detecting serum-derived EVs have hindered the clinical application of EVs in early CRC diagnosis.

**Methods:**

In the discovery phase, we conducted a comprehensive 4D-DIA proteomic analysis of serum-derived EVs samples from 37 individuals, performing an initial screening of EVs surface proteins. In the technical validation phase, we developed an extraction-free CRC-EVArray microarray to assess the expression of these potential EVs surface proteins in a multi-centre study comprising 404 individuals. In the application phase, the authors evaluated the diagnostic efficacy of the CRC-EVArray model based on machine-learning algorithms.

**Results:**

Through 4D-DIA proteomic analysis, the authors identified seven potential EVs surface proteins showing significantly differential expression in CRC patients compared to healthy controls. Utilizing our developed high-throughput CRC-EVArray microarray, we further confirmed the differential expression of three EVs surface proteins, FIBG, PDGF-β and TGF-β, in a large sample population. Moreover, we established an optimal CRC-EVArray model using the NNET algorithm, demonstrating superior diagnostic efficacy with an area under the curve (AUC) of 0.882 in the train set and 0.937 in the test set. Additionally, we predicted the functions and potential origins of these EVs-derived proteins through a series of multi-omics approaches.

**Conclusions:**

Our systematic exploration of surface protein expression profiles on serum-derived EVs has identified FIBG, PDGF-β, and TGF-β as novel diagnostic biomarkers for CRC. The development of CRC-EVArray diagnostic model based on these findings provided an effective tool for the large-scale CRC screening, thus facilitating its translation into clinical practice.

## Background

Highlights

- The 4D-DIA proteomics approach was employed to identify differential protein expression on the membrane of extracellular vesicles (EVs) derived from serum samples of colorectal cancer (CRC) patients compared with those from healthy controls.
- Employing our developed extraction-free high-throughput CRC-EVArray microarray, the differential expression of 3 EVs surface proteins, FIBG, PDGF-β and TGF-β, were further confirmed in both train and test sets.
- An optimal CRC-EVArray model was established to detect CRC based on a machine-learning algorithm, demonstrating superior diagnostic performance with an area under the curve (AUC) of 0.882 in the train set and 0.937 in the test set.

Colorectal cancer (CRC) is the third most prevalent cancer globally and concurrently represents the third leading cause of mortality among digestive system disorders^1^. The 5-year survival rate for stage I CRC patients is 90%, but drops significantly to only 10% for stage IV CRC patients^2^. Consequently, early screening for CRC is crucial for reducing mortality associated with this disease. CRC screening approaches can be broadly classified into two categories: invasive colonoscopy and non-invasive multi-target stool DNA testing^3^. However, the invasiveness and expensiveness of colonoscopy limited its applicability to specific populations^4^. Moreover, multi-target stool DNA testing faced criticism due to its relatively high false-positive rate^5^. Haematological biomarkers, such as CEA and CA19-9, held the advantages of convenient sample collection and facilitation for dynamic monitoring^6^. However, the performance of conventional blood tumour biomarkers in clinical practice requires further enhancement.

Extracellular vesicles (EVs) have emerged as prominent players in liquid biopsy, capturing the spotlight in biomedical research^7^. Extensive studies have revealed that tumour cells actively engage in communication with immune cells within the microenvironment through EVs secretion, thereby facilitating tumour growth and metastasis^8,9^. In turn, these immune cells also released EVs, further fuelling tumour progression^9^. Excitingly, EVs were abundantly present in body fluids such as blood, urine, and bile, offering real-time updates on the dynamic landscape of various malignancies^10^. Consequently, the diverse cargo components carried by EVs, including RNA, DNA, proteins, and metabolites, have become potential diagnostic and prognostic biomarkers. However, it was vital to note that current research primarily focused on exploring the role of non-coding RNA in EVs-mediated liquid biopsy of tumours, including CRC, while the significance of EVs-derived protein components has been relatively overlooked. A comprehensive understanding of the pivotal role played by EV-associated proteins in liquid biopsy holds tremendous promise for advancing the diagnosis and prognosis of CRC.

Protein biomarkers derived from EVs often exhibit extremely low levels of expression, requiring the development of highly sensitive detection methods. Conventional techniques such as ELISA or western blotting typically require large volumes of body fluid (>2 ml) to achieve sufficient concentrations^11^. Moreover, due to the complexity of body fluids, time-consuming ultracentrifugation steps were often employed for the isolation and purification of EVs. The surface proteins of EVs were localized on the membrane, and their specific localization enabled direct detection through signal amplification via biosensors^12^. However, recently developed biosensors, such as thermally induced luminescent sensors, nano-photonics EVs sensors, and micro-nuclear magnetic resonance, posed critical challenges for clinical applications due to their reliance on expensive equipment or intricate sensing protocols^13–16^. Furthermore, these biosensors could not overcome the limitation of requiring large sample volumes. Encouragingly, we have established a method to directly detect EVs membrane proteins in plasma to predict the immunotherapeutic response in gastric cancer patients in 2022^17^. This platform exhibited high sensitivity and required simple instrumentation, by which only 10 μl serum was needed for the assay. While building upon this platform, we further developed a novel microarray chip named CRC-EVArray, which was specifically designed for CRC diagnosis in the present study.

**Figure 1:**
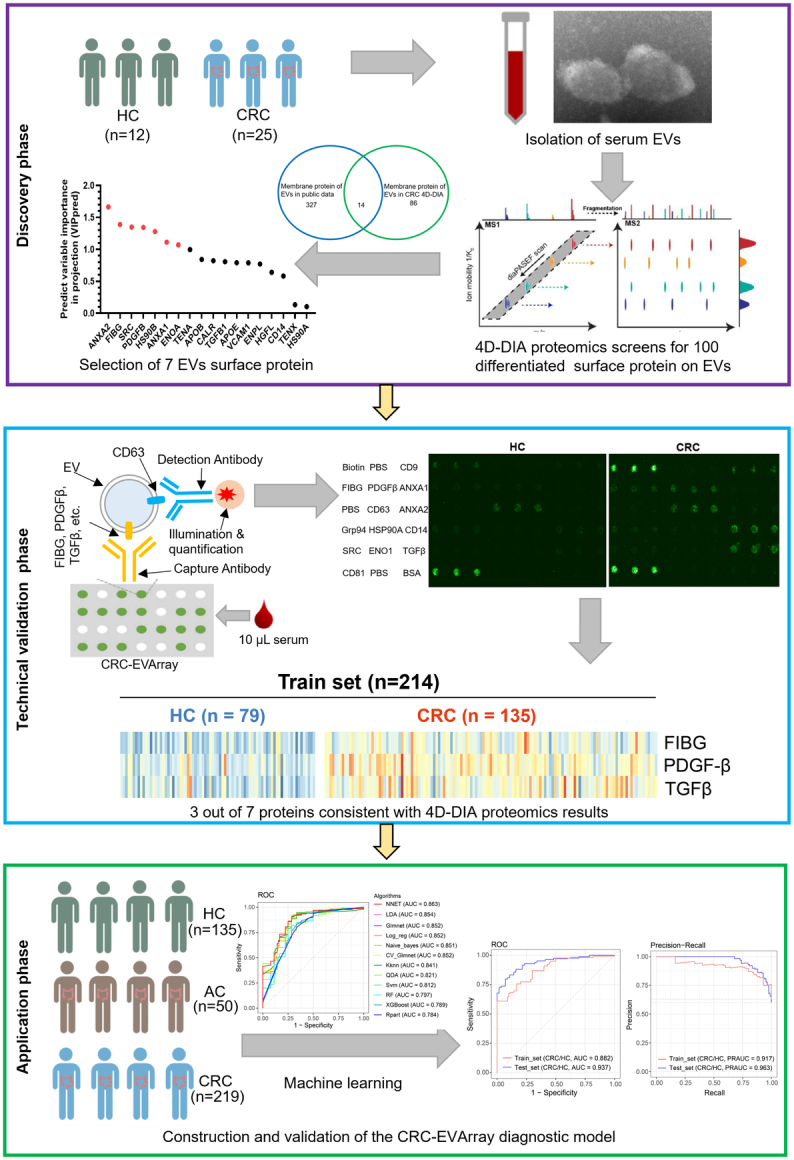
A general workflow for identification and validation of surface proteins on serum-derived extracellular vesicles (EVs) for colorectal cancer (CRC) diagnosis. HC, healthy control.

In this study, we aimed to evaluate the potential value of surface proteins derived from serum EVs as novel biomarkers for screening and diagnosing CRC. We employed 4D-DIA proteomics technology for the initial selection of target proteins (Fig. 1). Subsequently, we developed a CRC-EVArray microarray platform to provide a robust tool for high-throughput surface protein assays of EVs and validated the diagnostic efficacy of the combination of FIBG, PDGF-β, TGF-β, and CEA in a larger sample population using machine-learning (ML) algorithms.

## Methods

### Human serum samples

The discovery set consisted of 12 healthy controls (HC) and 25 patients with CRC. Serum samples from HC and CRC patients were collected from Centre A between May 2022 and June 2022.

The train set was composed of 79 HC, 25 patients with advanced adenoma (AA), and 135 patients with CRC. Serum samples were collected from Centre A between August 2020 and March 2022.

The test set consisted of 56 HC, 25 patients with AA, and 84 patients with CRC. Serum samples were collected from Centre B between September 2020 and December 2022.

This was a retrospective, cross-sectional case-control study. The enroled HC had no history of intestinal diseases, inflammatory diseases, or any other significant medical conditions and had undergone a normal colonoscopy. The diagnosis of CRC was confirmed through histopathological examination, and serum samples were collected at the time of diagnosis prior to tumour resection and chemoradiotherapy. The definition of AA was based on the presence of high-grade dysplasia, a villous component, or a lesion size greater than 1 cm. Individuals with a family history of familial adenomatous polyposis or hereditary nonpolyposis CRC, as well as those who had undergone previous colonic surgery prior to CRC diagnosis, were excluded from the study. The participants’ age ranged from 18 to 90 years and the gender ratio was roughly equal between male and female participants. Informed consent was obtained from all participants. The clinical characteristics of these individuals were described in Supplemental Table 1, Supplemental Digital Content 1, http://links.lww.com/JS9/C460.

### Serum-derived EVs isolation

To isolate serum-derived EVs, 1 mL fresh serum was centrifuged at 4×10^3^ *g* for 20 min to remove cellular components. To further eliminate apoptotic bodies, the supernatant was centrifuged at 1.2×10^4^ *g* for 20 min and purified through a 0.22 µm filter. The filtered supernatant was then ultracentrifuged at 1.2×10^5^ *g* for 90 min using an OptimaTM XE ultracentrifuge (Beckman Coulter). The resulting pellet was collected by pipetting the supernatant, resuspended in PBS, and subjected to an additional centrifugation at 1.2×10^5^ *g* for 90 min. After careful removal of the supernatant, the isolated EVs samples were suspended in 100 μl of PBS and added to the mini-EV purification column to remove free proteins and nucleic acids by adsorption. The remaining suspension represented high-purity purified EVs and was stored at −80°C for further use.

### Transmission electron microscopy (TEM)

A 10 μl droplet of EVs suspension was placed on a copper grid with a carbon film for 30 minutes. The EVs were then fixed and incubated with anti-FIBG (Invitrogen, PA5-29734), anti-PDGF-β (Servicebio, GB11261), or anti-TGF-β (Servicebio, GB11179) at 4°C overnight. Subsequently, the grids were incubated with 10 nm-gold labelled secondary antibody for 120 min at room temperature. After rinsing with 0.1% BSA in PBS, the grids were fixed in 2.5% glutaraldehyde for 20 min. Finally, the grids were washed with PBS and distilled water, followed by contrast staining using 2% phosphotungstic acid staining solution. The cuprum grids were then imaged using TEM HT7800 (Hitachi).

### Western blotting

Western blotting was performed according to a standard protocol, as previously described^18^. The following primary antibodies were used: Grp94 (Servicebio, GB111280), Calnexin (Servicebio, GB111369), Calnexin (Beyotime, AF1471), and TSG101 (Beyotime, AF8259).

### Nanoparticle tracking analysis (NTA)

The EVs samples were diluted with PBS and detected using a ZetaVIEW S/N 21-734 nanoparticle tracking analyzer (Particle Metrix, Munich, Germany).

### 4D-DIA quantitative proteomic

4D-DIA quantitative proteomic analysis was conducted by Shanghai Genechem Co., Ltd. A total of 37 EVs samples from the discovery cohort were subjected to protein extraction via ultrasonic lysis, in combination with 1% protease inhibitor. The protein samples were subjected to a two-step digestion process: overnight digestion at a 1:50 trypsin to protein mass ratio, followed by 4 h digestion at a 1:100 trypsin to protein mass ratio. The resulting peptides were desalted using a Strata X C18 solid-phase extraction column and then vacuum-dried. The resulting MS/MS data were processed using the MaxQuant search engine (version 1.5.2.8).

### CRC-EVArray

Beijing EVbio Technology Co., Ltd. assisted us in the construction of the CRC-EVArray, which was produced and generated as previously described^17^. In brief, all the antibodies were diluted in PBS with 5% glycerol and then printed onto a 14-well setup 3D modified slide surface (75.6×25.0 mm, Capital Biochip Corp) in triplicate at a concentration of 200 µg/ml using a microarrayer (Arrayjet). The temperature and humidity were maintained at 15–18°C and 55–65%, respectively. The following primary antibodies were used for capture: CD9 (Ancell, 156-020), FIBG (Proteintech, 66186-1-Ig), PDGF-β (Sinobiological, 102425-T32), ANXA1 (Proteintech, 66344-1-Ig), CD63 (Ancell, 215-820), ANXA2 (Proteintech, 66035-1-Ig), HSP90-β (Proteintech, 11405-1-AP), CD14 (Sinobiological, 10073-MM01), SRC (Proteintech, 60315-1-Ig), ENO1 (Sinobiological, 11554-R063), TGF-β (Sinobiological, 10804-R016), and CD81 (Ancell, 302-820). PBS with 5% glycerol served as the negative control, while 100 μg/ml biotinylated BSA served as the positive control. Following blocking, the serum samples were diluted 1:10 with 10 μl in washing buffer at room temperature and then incubated overnight at 4°C. After washing, the slides were incubated with the biotinylated detection antibody anti-CD63 diluted 1:1500 in the washing buffer. For detection, Cy3-labelled streptavidin was added. After 60 min of incubation, the slides were washed and scanned using a GenePix 4000A microarray scanner (Molecular Devices). The fluorescent signals were extracted using a GenePix Pro image analysis software (Molecular Devices). The signal intensity of each antibody was calculated by subtracting the mean of the negative triplicate. To evaluate the protein density on the EVs, normalization of each spot signal was performed. CD63 was employed as the normalization factor. For each antibody, the signal intensity was divided by the normalization factor before further analysis. CRC-EVArray samples were tested in random order, and the experimental operators were blinded to the grouping of patients.

### Machine-learning and development of the CRC-EVArray diagnostic model

Orthogonal Partial Least Squares Discriminant Analysis (OPLS-DA) was conducted via SIMCA software version 14.1. Proteins exhibiting a Predictive Variable Importance in projection greater than 1 (VIPpred>1) were considered potential EVs biomarkers for CRC discrimination. After validating the CRC-EVArray microarray, 3 out of the 7 proteins were selected for further modelling. Subsequent ML models were conducted by using mlr3 R package. A total of 12 ML algorithms were employed to establish diagnostic models for optimal algorithm selection. Utilizing the optimal NNET algorithm, diverse variables, including FIBG, PDGF-β, TGF-β, CEA, and CA19-9, were incorporated into the diagnostic model. The final CRC-EVArray diagnostic mode, based on the most effective variable combination, was determined through superior diagnostic efficiency assessment. To ensure model reliability, the train set was employed to validate the diagnostic performance via confusion matrix, receiver operating characteristics (ROC) curve, and precision-recall (PR) curve.

### Bioinformatics analysis

The RNA-seq data and clinical data from the TCGA CRC database were sourced from The Cancer Genome Atlas (TCGA) databases. Gene Set Enrichment Analysis (GSEA) was employed to predict Kyoto Encyclopedia of Genes and Genomes (KEGG) gene sets of the Molecular Signature Database v7.4 based on the FIBG, PDGF-β or TGF-β high and low expressed phenotype. The EnrichmentMap plugin in Cytoscape 3.8.2 software was utilized to establish associations within enriched pathways. Leading edge analysis, performed through GSEA 4.1.0, revealed pivotal genes involved in the EnrichmentMap pathway network. Raw single-cell RNA sequencing (scRNA-seq) data were acquired from the Gene Expression Omnibus (GEO) database (GSE132465 and GSE178341).

### Statistical analysis

Statistical analyses were conducted using IBM SPSS Statistics 25.0. The data variability was presented as the mean ± SD and analyzed using an unpaired Student’ s *t*-test between two groups for normally distributed data. Otherwise, the data were analyzed via the nonparametric Mann–Whitney test. The diagnostic performance metrics, including area under the curve (AUC), precision-recall AUC (PRAUC), classification error (CE), sensitivity, specificity, precision, recall, accuracy, and F1 score, were calculated using the mlr3 R package. *P* less than 0.05 was defined statistical significance.

## Results

### Discovery of differentially expressed surface proteins on serum-derived EVs in CRC patients using 4D-DIA proteomics

**Figure 2:**
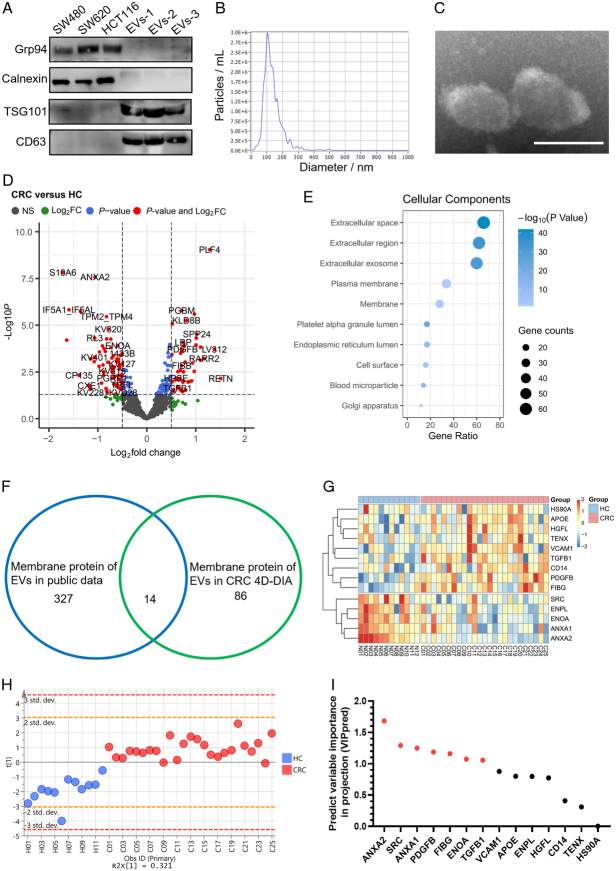
Discovery of differentially expressed surface proteins on serum-derived extracellular vesicles (EVs) in colorectal cancer (CRC) patients using 4D-DIA proteomics. (A) Detection of EVs markers TSG101 and CD63 in isolated serum EVs by western blotting analysis. (B) Representative diagram of sizes and distribution of isolated EVs by nanoparticle tracking analysis. (C) Representative transmission electron microscopy image showing the morphology of EVs isolated from serum. Scale bar=100 nm. (D) The volcano plots depicting the differentially expressed proteins (DEPs) in serum EVs. (E) GO functional analysis of the DEPs in serum EVs. (F) Venn diagram illustrating the overlap between membrane proteins of EVs identified in 4D-DIA proteomics and public data. (G) Heatmap displaying the common dysregulated surface proteins in proteomics. (H) Scatter plot demonstrating the discrimination of surface proteins on serum-derived EVs between CRC and healthy control subjects through orthogonal partial least squares discriminant analysis. (I) Selection of 7 candidate proteins based on their VIPpred scores>1.

The first object of this study was to comprehensively characterize surface proteins presented on EVs in the serum of CRC patients. To achieve this goal, we initially isolated EVs from serum samples using ultracentrifugation. Western Blotting analysis confirmed that the isolated EVs robustly expressed EV-specific biomarkers, as compared to those of CRC cell lines (Fig. 2A). The NTA results revealed that the diameter of the isolated EVs predominantly ranged from 50 to 200 nm (Fig. 2B). Additionally, TEM provided visual evidence of isolated EVs’ morphology (Fig. 2C). These findings indicated the successful isolation of highly pure EVs from serum samples. Subsequently, we conducted 4D-DIA quantitative proteomic assays on these isolated EVs, enabling the screening of 166 differentially expressed proteins (DEPs) by comparing CRC patients and HC within the discovery cohort (Fig. 2D). Notably, Gene Ontology (GO) analysis highlighted that 100 DEPs were potentially localized to the membrane (Fig. 2E). Through intersection with 341 previously reported EVs surface proteins from the literature^19–23^, 14 surface proteins were further screened (Fig. 2F). A heatmap was generated to demonstrate the expression patterns of these 14 surface proteins in each individual patients in the discovery cohort (Fig. 2G). Using orthogonal partial least squares discriminant analysis (OPLS-DA), we further determined the discriminatory potential of the 14 selected candidate proteins in distinguishing CRC based on the 4D-DIA proteomics data (Fig. 2H; Supplemental Fig 1A, Supplemental Digital Content 2, http://links.lww.com/JS9/C461). Furthermore, VIPpred analysis identified 7 out of the 14 proteins as candidate EVs proteins, namely ANXA2, SRC, ANXA1, PDGFB, FIBG, ENOA, and TGFB1 (Fig. 2I). These proteins were found to be the core contributors to differentiate CRC patients from HC.

### Validation of FIBG, PDGF-β, and TGF-β as upregulated surface proteins of serum-derived EVs in CRC patients

**Figure 3:**
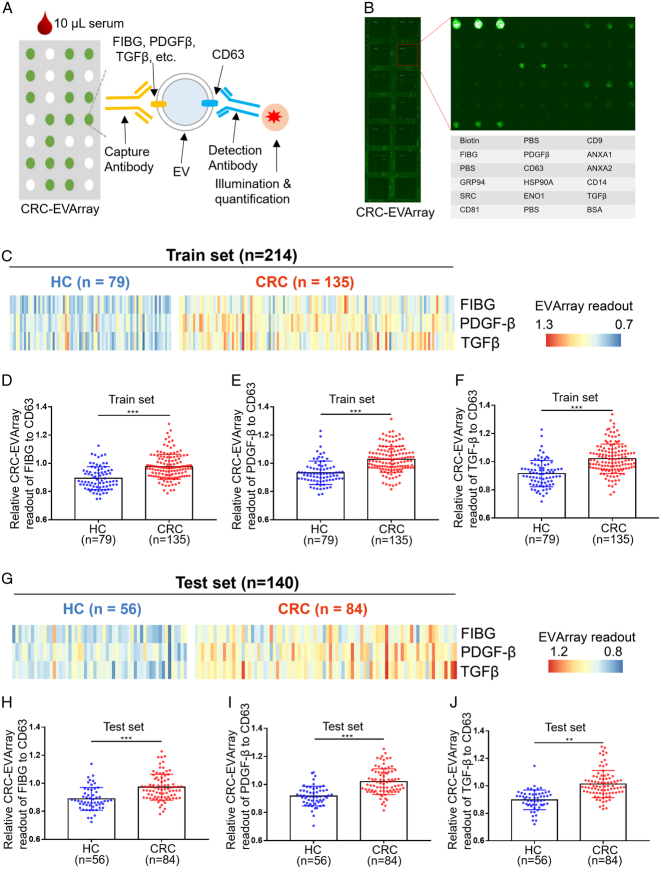
Validation of FIBG, PDGF-β, and TGF-β as upregulated surface proteins of serum-derived extracellular vesicles (EVs) in colorectal cancer (CRC) patients. (A) Schematic representation of the CRC-EVArray microarray used for the detection of candidate surface proteins on serum-derived EVs. (B) Representative fluorograms showing the CRC-EVArray assays. (C) Heatmaps displaying the relative CRC-EVArray readouts of serum samples from HC and CRC patients in the train set. (D–F) Relative CRC-EVArray readouts of FIBG (D), PDGF-β (E), and TGF-β (F) in the train set. ***P<0.001; *P* values from unpaired *t*-test are shown. (G) Heatmaps presenting the relative CRC-EVArray readouts of serum samples from HC and CRC patients in the test set. (H–J) Relative CRC-EVArray readouts of FIBG (H), PDGF-β (I), and TGF-β (J) in the test set. ***P<0.001; **P<0.01; *P* values from unpaired *t*-test are shown.

Subsequently, we evaluated large-scale samples to validate the findings of the proteomic analyses. Given the lack of convenient tools for detecting surface proteins on EVs, we innovated the CRC-EVArray microarray chip based on the principles elucidated in a previously published article by our collaborative team^17^. This pioneering chip facilitated the simultaneous detection of 7 surface proteins on EVs, requiring only 10 μl serum. Figure 3A, B illustrated the schematic diagram and physical appearance of CRC-EVArray. The fluorescence intensity of commonly employed EV-specific biomarkers (CD9, CD63 and CD81) in the HC group did not differ significantly between the train and test sets, confirming the data stability of the CRC-EVArray platform (Supplemental Fig 1B-E). Notably, recent literature has suggested CD63 served as a more appropriate internal reference for EVs^24^. Consequently, after quantifying the CRC-EVArray signal intensity, CD63 molecular correction was employed to obtain the final expression readout for each surface protein (Fig. 3C-J). Figure 3C and G summarize the relative CRC-EVArray readout for the three subpopulations of serum-derived EVs in the train and test sets, respectively. We observed significant elevation exclusively in the CRC group for FIBG, PDGF-β, and TGF-β, which aligned consistently with the proteomic results (Fig. 3C-J). While the results for ANXA1, ANXA2, SRC, and ENO1 in the clinical cohorts did not correspond with the proteomic findings (Supplemental Fig 2, Supplemental Digital Content 3, http://links.lww.com/JS9/C462). Taken together, we confirmed the aberrant expression of surface proteins FIBG, PDGF-β, and TGF-β on serum-derived EVs in CRC patients through rigorous analysis of a large-scale sample cohort.

### Analysis the relationship between the expression levels of FIBG, PDGF-β, and TGF-β on serum-derived EVs and the clinicopathological characteristics of CRC patients

**Figure 4:**
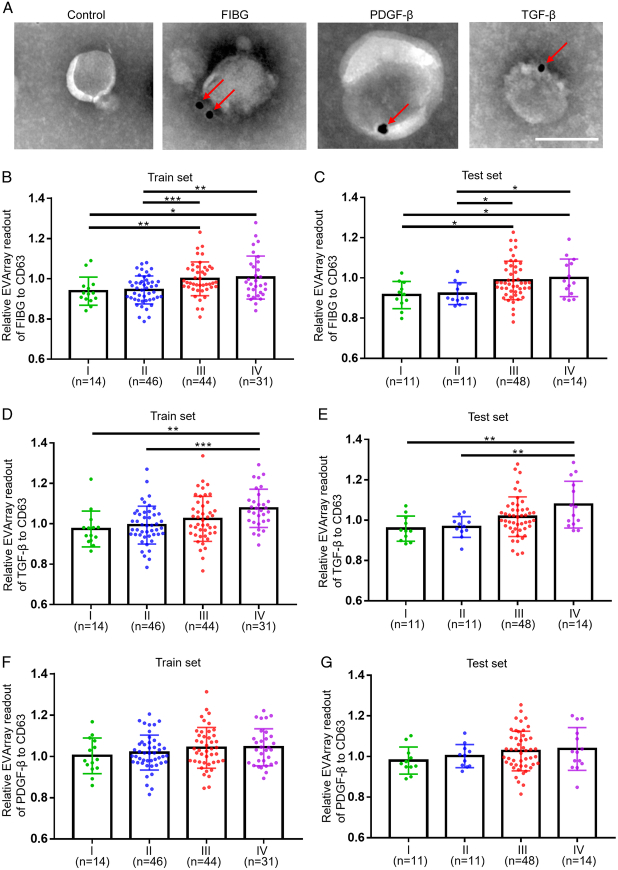
Analysis the relationship between the expression levels of FIBG, PDGF-β, and TGF-β on serum-derived extracellular vesicles (EVs) and the clinicopathological characteristics of colorectal cancer (CRC) patients. (A) Representative transmission electron microscopy diagram showing immunogold-labelled FIBG, PDGF-β, and TGF-β on EVs. Scale bar=100 nm. (B, D, F) Relative levels of FIBG (B), TGF-β (D), and PDGF-β (F) on EVs across CRC stages (stage I, *n*=14; stage II, *n*=46; stage III, *n*=44; stage IV, *n*=31) in the train set. ***P<0.001; **P<0.01; *P<0.05; *P* values from unpaired *t*-test are shown. (C, E, G) Relative levels of FIBG (C), TGF-β (E), and PDGF-β (G) on EVs across CRC stages (stage I, *n*=11; stage II, *n*=11; stage III, *n*=48; stage IV, *n*=14) in the test set. **P<0.01; *P<0.05; *P* values from unpaired *t*-test are shown.

We next employed immunogold TEM to further confirm the presence of FIBG, PDGF-β, and TGF-β on the surface of serum-derived EVs visually. Remarkably, distinct clusters of gold nanoparticles were observed surrounding EVs, specifically labelled with anti-FIBG, PDGF-β, and TGF-β immunogold tags, providing compelling visual evidence of their expression on EVs (Fig. 4A). Upon analyzing the clinicopathological features of CRC patients, we observed that the expression levels of FIBG and TGF-β on serum-derived EVs were positively correlated with both clinical stages (Fig. 4B, E; Supplemental Tables 2 and 3, Supplemental Digital Content 1, http://links.lww.com/JS9/C460). Specifically, FIBG expression exhibited a positive correlation with T stage in both the train and test sets (Supplemental Table 2, Supplemental Digital Content 1, http://links.lww.com/JS9/C460). Additionally, TGF-β expression displayed an association with the M stage, indicating its potential as a predictor of distant metastasis in CRC patients (Supplemental Table 3, Supplemental Digital Content 1, http://links.lww.com/JS9/C460). Conversely, PDGF-β expression did not display any significant correlation with these clinical indicators, although it did demonstrate an association with differentiation in the train cohort (Fig. 4F-G; Supplemental Table 4, Supplemental Digital Content 1, http://links.lww.com/JS9/C460). Collectively, these findings emphasized the intricate link between the surface proteins FIBG, PDGF-β, and TGF-β on EVs and the clinicopathological characteristics of CRC patients.

### Construction and validation of the CRC-EVArray diagnostic model for CRC detection

**Figure 5:**
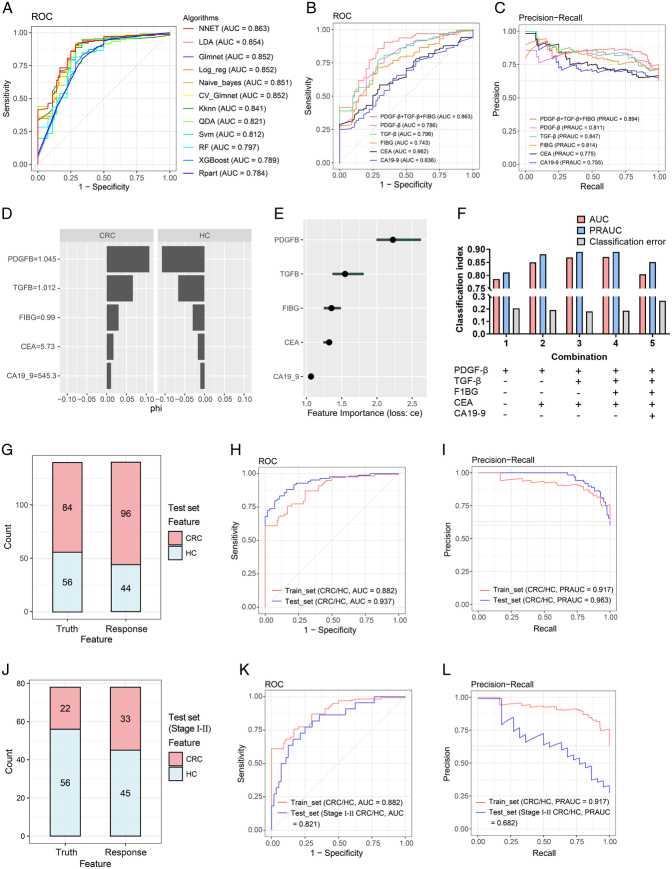
Construction and validation of the CRC-EVArray diagnostic model for colorectal cancer (CRC) detection. (A) Receiver operating characteristics (ROC) curves illustrating the combined value of FIBG, PDGF-β, and TGF-β in ML diagnostic models employing diverse algorithms. (B, C) ROC curve (B) and precision-recall (PR) curve (C) of the Neural Network (NNET) diagnostic models based on the indicated variables within the train set. (D) Shapley value plots depicting the impact of FIBG, PDGF-β, TGF-β, CA19-9, and CEA on discriminating colorectal cancer (CRC) patients from healthy control (HC). (E) Variable importance score plot showcasing the contribution of the 5 variables in the NNET diagnostic model. (F) Area under the curves (AUC), precision-recall AUC (PRAUC), and classification error (CE) values of the NNET diagnostic models with different variable combinations. (G) Confusion matrix presenting the prediction outcomes for 140 untrained sample in the test set using the CRC-EVArray diagnostic model. (H, I) ROC curve (H) and PR curve (I) plotted for the CRC-EVArray diagnostic model in both the train and test sets. (J) Confusion matrix demonstrating the prediction results for 22 stage I–II CRC patients and 56 HC sample in the test set using the CRC-EVArray diagnostic model. (K, L) ROC curve (K) and PR curve (L) of the CRC-EVArray diagnostic model for stage I–II CRC patients.

In pursuit of the most informative variables, we formulated ML diagnostic models centred around FIBG, PDGF-β, and TGF-β. Among the array of 12 distinct ML algorithms explored, the Neural Network (NNET) model emerged as the optimal choice, displaying superior performance metrics encompassing AUC, PRAUC, and classification error (CE) (Fig. 5A; Supplemental Fig 3A, B, Supplemental Digital Content 4, http://links.lww.com/JS9/C463; Supplemental Table 5, Supplemental Digital Content 1, http://links.lww.com/JS9/C460). Consequently, the NNET algorithm was selected as the foundation for the subsequent ML models development. Through a comprehensive evaluation of FIBG, PDGF-β, and TGF-β within the framework of the NNET diagnostic model, remarkable diagnostic efficacy was unveiled, surpassing that of traditional CRC biomarkers CEA and CA19-9 (Fig. 5B, C). The ROC curves distinctly exhibited heightened AUC values for FIBG (AUC: 0.743), PDGF-β (AUC: 0.786), and TGF-β (AUC: 0.796), outperforming CEA (AUC: 0.662) and CA19-9 (AUC: 0.636) within the train set (Fig. 5B). Notably, the combination of FIBG, PDGF-β, and TGF-β yielded a remarkable AUC of 0.863 and an elevated PRAUC of 0.894 (Fig. 5B, C). Profound Accumulated Local Effects (ALE) analysis substantiated the superior predictive impact of FIBG, PDGF-β, and TGF-β in CRC prognosis, transcending the predictive power of CEA and CA19-9 (Supplemental Fig 3C, Supplemental Digital Content 4, http://links.lww.com/JS9/C463). The Shapley value analysis concurred, highlighting the substantial contribution of elevated PDGF-β (≥ 1.045), TGF-β (≥ 1.012), and FIBG (≥ 0.99) levels in discerning CRC from HC, corroborating the results of the importance analysis (Fig. 5D, E).

Aiming for optimal integration of novel EVs-derived biomarkers and traditional CRC indicators, different NNET diagnostic models rooted in diverse variable combinations were constructed (Supplemental Table 6, Supplemental Digital Content 1, http://links.lww.com/JS9/C460). As depicted in Fig. 5F, the combination of FIBG, PDGF-β, TGF-β, and CEA yielded the most exceptional diagnostic performance. After hyperparameter tuning, the finely tuned diagnostic model founded upon the 4 variables was eventually named the CRC-EVArray diagnostic model, characterized by an AUC of 0.882, PRAUC of 0.917, and CE of 0.186 (Supplemental Fig 3D, Supplemental Digital Content 4, http://links.lww.com/JS9/C463; Supplemental Table 7, Supplemental Digital Content 1, http://links.lww.com/JS9/C460). Its remarkable prowess was further validated through rigorous testing within the independent set, as attested by the confusion matrix, which showcased an impressive accuracy of 0.829 (Fig. 5G). The model’s exceptional diagnostic acumen was unequivocally confirmed by the ROC and PRAUC curves, registering an AUC of 0.937 and a PRAUC of 0.963 (Fig. 5H, I). Remarkably, outcomes of the confusion matrix analysis underscored the robust accuracy of the CRC-EVArray diagnostic model in distinguishing stage I–II CRC patients from HC (Fig. 5J). This compelling discriminative power was reinforced by the ROC and PRAUC curves, which firmly established the diagnostic efficacy of the CRC-EVArray model for early-stage CRC (Fig. 5K, L; Supplemental Table 7, Supplemental Digital Content 1, http://links.lww.com/JS9/C460).

### Comparison of FIBG, PDGF-β, and TGF-β levels on serum-derived EVs between HC and AA patients

**Figure 6:**
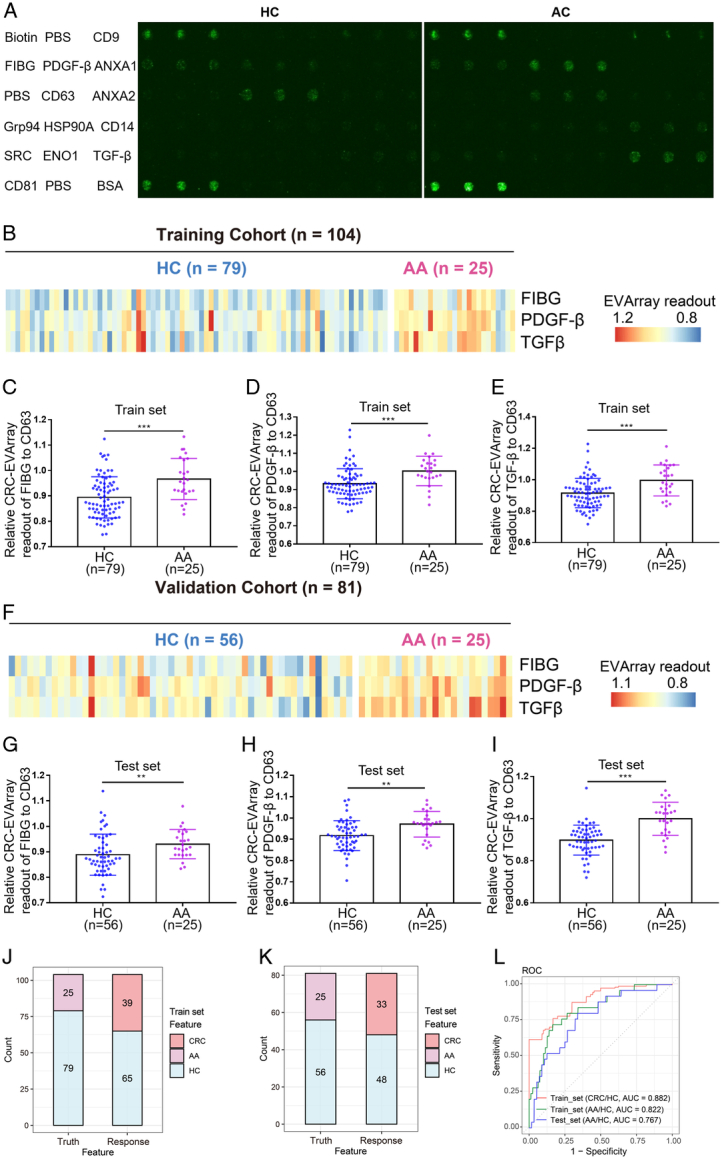
Comparison of FIBG, PDGF-β, and TGF-β levels on serum-derived EVs between healthy control (HC) and advanced adenoma (AA) patients. (A) Representative fluorograms showing the colorectal cancer (CRC)-EVArray assays of serum samples from HC and AA patients. (B) Heatmaps displaying the relative CRC-EVArray readouts of serum samples from HC and AA patients in the train set. (C–E) Relative CRC-EVArray readouts of FIBG (C), PDGF-β (D), and TGF-β (E) in the train set. ***P<0.001; *P* values from unpaired *t*-test are shown. (F) Heatmaps presenting the relative CRC-EVArray readouts of serum samples from HC and AA patients in the test set. (G–I) Relative CRC-EVArray readouts of FIBG (G), PDGF-β (H), and TGF-β (I) in the test set. ***P<0.001; **P<0.01; *P* values from unpaired *t*-test are shown. (J, K) Confusion matrix showing the predicted results of the CRC-EVArray diagnostic model identifying AA as CRC in the test set (J) and test set (K). (L) Receiver operating characteristics (ROC) curve of the CRC-EVArray diagnostic model for AA patients. Data were presented as mean ± SD; **P<0.01, and ***P<0.001.

Conventional non-invasive techniques have exhibited limited efficacy in the timely detection of CRC, with even more pronounced challenges in detecting AA. To address this pressing concern, we collected serum samples from AA patients and utilized the CRC-EVArray platform to investigate whether aberrant expression of surface proteins on EVs could offer a non-invasive means of detection during this critical window (Fig. 6A). The relative CRC-EVArray readouts pertaining to the distinct subgroups of serum-derived EVs from AA patients in the train set were summarized in Fig. 6B. Strikingly, our observation revealed a conspicuous upsurge in the levels of EVs surface proteins, FIBG, PDGF-β, and TGF-β, within AA patients (Fig. 6C, E). This intriguing finding was further validated with the relative readouts obtained from the 3 subpopulations of EVs in the independent test set, mirroring the outcomes of the train set (Fig. 6F, I). Impressively, the CRC-EVArray model showcased notable diagnostic capability in effectively discriminating between HC and AA patients, yielding an AUC of 0.822 in the train set and 0.767 in the test set, respectively (Fig. 6J, L). Taken together, our findings underscored the promising potential of the CRC-EVArray model as a robust tool for the early detection of CRC and AA.

### Functional prediction and potential origins of EVs-derived FIBG, PDGF-β, and TGF-β

**Figure 7:**
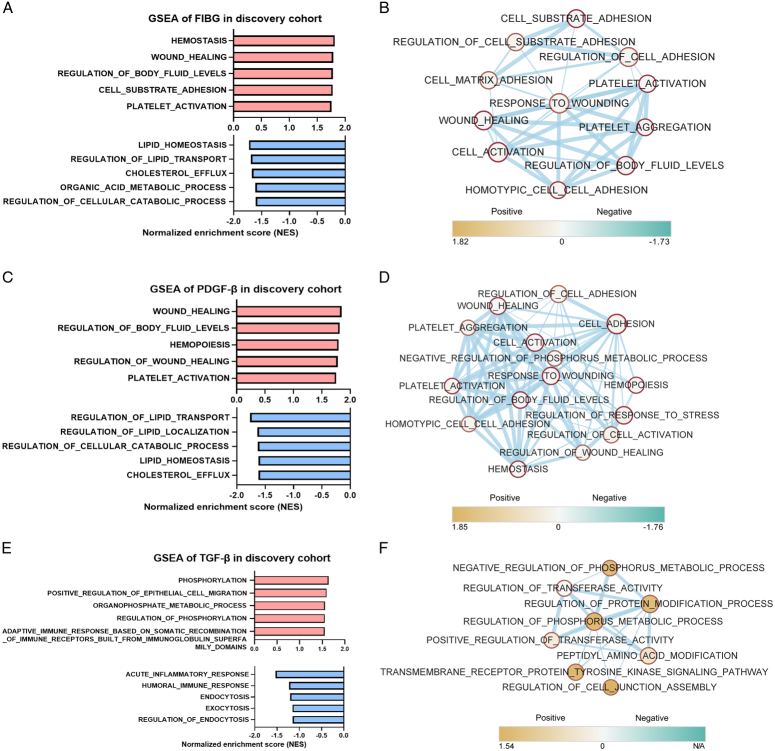
Functional prediction of extracellular vesicles (EVs)-derived FIBG, PDGF-β, and TGF-β. (A) Gene Set Enrichment Analysis (GSEA) displaying the top-ranking pathways based on EVs-derived FIBG-high (red) and -low (blue) phenotypes. (B) EnrichmentMap network analysis of associated pathways enriched in the EVs-derived FIBG-high phenotype. (C) GSEA displayed the top-ranking pathways based on EVs-derived PDGF-β-high (red) and -low (blue) phenotypes. (D) EnrichmentMap network analysis of associated pathways enriched in the EVs-derived PDGF-β-high phenotype. (E) GSEA displayed the top-ranking pathways based on EVs-derived TGF-β-high (red) and -low (blue) phenotypes. (F) EnrichmentMap network analysis of associated pathways enriched in the EVs-derived TGF-β-high phenotype.

To gain insight into the potential roles of EVs-derived FIBG, PDGF-β, and TGF-β in CRC, GSEA was meticulously executed. Our findings unveiled that within the discovery set, EVs-derived FIBG exhibited enrichment in pathways linked to wound healing, cell-substrate adhesion, and platelet activation, indicative of their probable functional involvement (Fig. 7A). Meanwhile, a negative correlation with lipid homoeostasis and transport pathways was also observed (Fig. 7A). Employing EnrichmentMap analysis, we further examined the intricate associations encompassing these enriched terms, disclosing robust connections between wound healing and platelet activation (Fig. 7B). Interestingly, the functional insights derived from EVs-derived PDGF-β closely paralleled those of FIBG, with the PDGF-β high-expression phenotype mirroring significant correlations with wound healing, cell adhesion and platelet activation (Fig. 7C, D). Parallel analyses were conducted for EVs-derived TGF-β, revealing its enrichment not only in processes like phosphorylation, cell migration, and metabolic processes but also underscoring its involvement in immune responses (Fig. 7E). These functional associations were intricately represented within the EnrichmentMap network (Fig. 7F).

**Figure 8:**
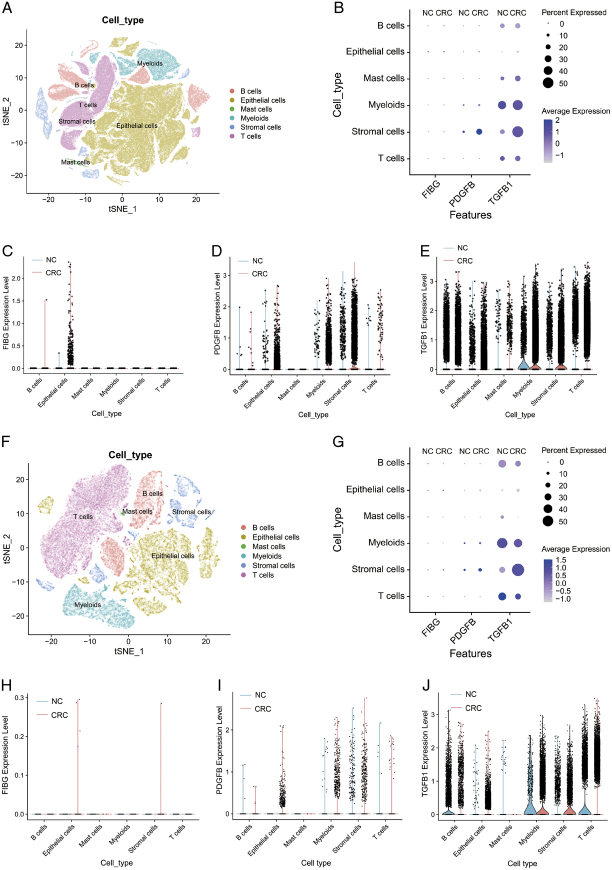
scRNA-seq analysis revealing the potential origins of extracellular vesicles (EVs)-derived FIBG, PDGF-β, and TGF-β. (A) t-SNE (t-distributed Stochastic Neighbor Embedding) plot illustrating distinct cell types in colorectal cancer (CRC) and normal tissues through scRNA-seq analysis from the GSE132465 dataset. (B) The expression of PDGF-β, TGF-β, and FIBG in normal and CRC tissues from the GSE132465 dataset. (C–E) Violin plot displaying the expression of PDGF-β (C), TGF-β (D), and FIBG (E) in normal and CRC tissues from the GSE132465 dataset. (F) t-SNE plot depicting distinct cell types in CRC and normal tissues through scRNA-seq analysis from the GSE178341 dataset. (G) The expression of PDGF-β, TGF-β, and FIBG in normal and CRC tissues from the GSE178341 dataset. (H–J) Violin plot presenting the expression of PDGF-β (H), TGF-β (I), and FIBG (J) from the GSE178341 dataset.

Meanwhile, we aimed to identify the specific cell types within CRC tumours responsible for packaging FIBG, PDGF-β, and TGF-β using single-cell transcriptomic data. A t-SNE plot delineated distinct cell populations within CRC and normal tissues by scRNA-seq analysis of the GSE132465 dataset (Fig. 8A; Supplemental Fig 4A, Supplemental Digital Content 5, http://links.lww.com/JS9/C464). Moderate increases of FIBG, PDGF-β and TGF-β expression were found in tumour epithelial cells. (Fig. 8B). FIBG appeared to be predominantly expressed within tumour epithelial cells, while PDGF-β and TGF-β exhibited broader distributions (Fig. 8C, E). Intriguingly, both PDGF-β and TGF-β presented aberrant elevation in the myeloid and stromal cells of CRC patients (Fig. 8D, E). The corroborative results from the scRNA-seq analysis of the GSE178341 dataset echoed these trends (Fig. 8F, J; Supplemental Fig 4B, Supplemental Digital Content 5, http://links.lww.com/JS9/C464). Collectively, we predicted the functions and potential origins of EVs-derived FIBG, PDGF-β, and TGF-β by a series of multi-omics approaches.

## Discussion

The transition from benign colorectal polyps to adenomas typically spanned a period of 5–10 years, during which tumour cells and affected stromal cells continuously released characteristic EVs, providing a valuable opportunity for the early diagnosis of CRC. Recent investigations have unveiled the association of the plasma EVs protein CD14 with the occurrence and prognosis of oesophageal squamous cell carcinoma, indicating its potential as a diagnostic and prognostic biomarker^25^. Furthermore, the combination of EVs proteins PIGF, FRIL, CRP, and FIBG demonstrated predictive abilities for assessing the risk of developing cholangiocarcinoma in individuals with primary sclerosing cholangitis^26^. However, the absence of clear targets for detecting serum-derived EVs has hindered the clinical application of EVs in early CRC diagnosis.

Notably, the surface of EVs was enriched with numerous membrane proteins, forming a dynamic corona that facilitated crosstalk between EVs and tumour microenvironments in 2022^23^. Our study focused on investigating proteins identified through 4D-DIA proteomics that were likely localized on the surface of EVs and 166 differentially expressed proteins were found between CRC patients and normal control. Consistent with our study, a prominent immune checkpoint molecule, programmed death-ligand 1 (PD-L1), have also been found localized on the surface of EVs^8^. Clinical studies revealed the expression level of EVs-derived PD-L1 could predict the immunotherapeutic response in gastric cancer and non-small cell lung cancer patients^27,28^. Animal model results demonstrated that PD-L1^+^ EVs secreted by melanoma and glioma cells suppressed the proliferation of CD8^+^ T cells and reduced lymphocyte infiltration in tumour tissues^29,30^. Moreover, PD-L1^+^ EVs released by tumour cells neutralized the efficacy of immune therapy by blocking PD-L1 antibodies^31^. Currently, there is a lack of precise predictive biomarkers for immunotherapy. Therefore, monitoring a range of immune checkpoint proteins on the surface of EVs might help identify more patients who could benefit from immunotherapy.

In recent years, significant progress has been made in next-generation biosensors for detecting proteins on the surface of EVs^32^. An approach utilized recombinase polymerase amplification technology, leveraging aptamers that specifically bound to membrane proteins on EVs^15,33^. This ingenious strategy converted protein signals into DNA signals and elucidated the predictive role of PD-L1^+^ EVs in the immunotherapeutic response of nasopharyngeal cancer patients^15^. Besides, another research group developed a microfluidic-based thermophoretic aptasensor, which detected the expression levels of eight EVs surface proteins in plasma to construct a diagnostic model for breast cancer^34^. However, these detection platforms were expensive, requiring specialized instruments or consumables. Moreover, the lack of specific aptamers for most surface proteins restricted the clinical applicability of these methods. In contrast, our developed CRC-EVArray platform offered a simple operation and allowed for high-throughput detection of multiple EVs surface proteins using just 10 µl blood. Through validation in multi-centre cohorts and assisted by ML algorithms, we identified FIBG/PDGF-β/TGF-β-enriched EVs exhibited excellent diagnostic performance for CRC. Factors such as patient inflammation, diet, and circadian rhythms had no impact on the reproducibility of the study results (Supplemental Fig 5, Supplemental Digital Content 6, http://links.lww.com/JS9/C465). Notably, this EVs signature demonstrated promising diagnostic efficacy for early-stage CRC patients, effectively compensating for the low sensitivity of multi-target DNA testing in stool sample. We are planning further evaluation of the potential of combining the CRC-EVArray diagnostic model with multi-target stool DNA testing as a solution for CRC screening.

Previous auxiliary diagnostic strategies have mainly focused on individual molecules of interest. However, tumours exhibited high heterogeneity, and the changes in a single molecule could not reveal the complete disease progression. We categorized CRC patients in the train and test sets into different subtypes based on pMMR/dMMR status, HER2 expression, and differentiation degree to evaluate the applicability of the diagnostic model across different CRC subtypes (Supplemental Fig 6, Supplemental Digital Content 7, http://links.lww.com/JS9/C466). These results suggested that further analysis of additional surface protein features might enable the identification of specific subtypes of CRC. With advancements in high-throughput omics technologies, more potential targets have been identified, leading to the increasing prevalence of multi-target diagnostics. In our study, which primarily delved into membrane proteomics, the integration of ML algorithms holds the potential to further enhance diagnostic efficacy through integrative multi-omics analysis. Similar to our study, the involvement of ML has significantly impacted the tumour diagnostic landscape, aiding in the identification of valuable information from vast amounts of data^35^. ML has been employed to enhance the diagnostic efficacy of 10 protein panels for pancreatic ductal adenocarcinoma, utilizing 78 differential proteins identified through serum proteomics screening in 2021^36^.

Traditionally, cytokines were believed to exist primarily in a soluble form within the body. However, recent studies have unveiled their presence on the surface of EVs, offering increased stability by shielding them from degradation in the surrounding environment^8,20^. For instance, VEGF on the surface of EVs exhibited enhanced resistance to enzymatic degradation, thereby extending its half-life and potentially contributing to drug resistance^37,38^. Similarly, TGF-β have been detected on the surface of EVs secreted by tumour cells, playing a role in tumour-associated fibroblast generation and the immune response^39^. Our bioinformatic analysis corroborated these findings, revealing the involvement of TGF-β^+^ EVs in immune response and their distribution among immune and tumour cells. Moreover, recent literature has reported that compounds such as melatonin, vitamin E, epicatechin, and fisetin could alleviate kidney damage by blocking the TGF-β pathway. Therefore, these compounds are also likely to have the potential to target TGF-β on the surface of EVs, reshaping the immune microenvironment and offering new strategies for tumour immunotherapy^40,41^. Besides, the cytokine PDGF-β has been documented on the surface of EVs, thereby inhibiting apoptosis of vascular smooth muscle cells in diabetic patients^42,43^. Additionally, in a stroke mouse model with blood-brain barrier dysfunction, EVs containing FIBGs released by peripheral blood-derived microglia were detected^44^. Studies have found that these FIBG-containing EVs can activate the activity of NLRP3 inflammasomes and induce lymphocyte aggregation, consistent with the characteristics of inflammation-carcinogenesis during the process of CRC development. These clues suggested that our constructed platform could not only be used for early screening of cancers but also for monitoring the progression of chronic diseases such as diabetes and stroke, allowing real-time dynamic assessment of patients’ physical condition.

Despite these promising findings, our study had several limitations that warranted consideration. A larger patient cohort were required to further validate the clinical efficacy of serum FIBG/PDGF-β/TGF-β-enriched EVs in CRC screening. The inclusion of patients with other gastrointestinal malignancies was essential to ascertain the specificity of this panel for CRC or its potential as a pan-cancer biomarker. Moreover, while our results indicated a correlation between FIBG and TGF-β expression in EVs and CRC staging, longitudinal follow-up studies were imperative to confirm these potential prognostic implications (Fig. 4). Finally, although our study has drawn insights into the function and origin of EVs proteins through bioinformatics analysis and literature review, more experimental studies were warranted to further elucidate their roles and underlying mechanisms.

## Conclusions

In summary, we established a high-performance detection platform targeting surface proteins of EVs and identified surface proteins FIBG, PDGF-β, and TGF-β on EVs as potential diagnostic biomarkers for CRC. Moreover, we used the NNET algorithm to build an optimal CRC-EVArray model based on these surface protein results, which showed excellent diagnostic results in early CRC. It is noteworthy that the CRC-EVArray assay only required blood testing without the need for EVs isolation, which held great promise for widespread and large-scale early cancer screening applications. Furthermore, our study traced the origins of these EVs through single-cell transcriptomes, supporting further biological research to achieve precise cancer treatment. Our work also paved the way for the development of novel diagnostic strategies based on EVs’ surface protein analysis for various malignancies in the future.

## Ethical approval

This study was approved by the Ethics Committee of The Seventh Affiliated Hospital of Sun Yat-Sen University (KY-2020-039-01) and Sun Yat-sen University Cancer Center (B2022-475-01).

## Consent

Written informed consent was obtained from the patient for publication of this case report and accompanying images. A copy of the written consent is available for review by the Editor-in-Chief of this journal on request.

## Sources of funding

This study was supported by the fund from the National Nature Science Foundation of China (82103346; 82202829; 82202985); General project of Shenzhen Science and Technology Innovation Commission (JCYJ20220530145003008); Guangdong Basic and Applied Basic Research Foundation (2021A1515110094; 2022A1515111199; 2022A1515111062; 2023A1515220107).

## Author contribution

Conceptualization: H.Z.J., and Y.H.F.; methodology: D.C.C. and Y.H.F.; data collection: H.G.R., T.J., Z.L.J., H.X.Y., M.Y.F., Q.L.M., and Z.N.F.; statistical analysis: H.Z.J. and Y.H.F.; funding acquisition: H.Z.J., X.J.Y., M.C.Q., and Y.H.F.; study supervision: X.S. and L.C.H. All authors reviewed the manuscript and approved the final revision.

## Conflicts of interest disclosure

The authors declare that they have no competing interests.

## Research registration unique identifying number (UIN)

This study was registered in the http://www.chictr.org.cn/index.aspx, and UIN is ChiCTR2000034458(https://www.chictr.org.cn/showproj.html?proj=55633).

## Guarantor

Corresponding author Haofan Yin is the Guarantor.

## Data availability statement

**Availability of data and materials:** All data generated or analyzed during this study are included in this published article and its supplementary information. The data that support the findings of this study are available from the corresponding author, Haofan Yin, upon reasonable request.

## Provenance and peer review

Not applicable.

## Supplementary Material

**Figure:**
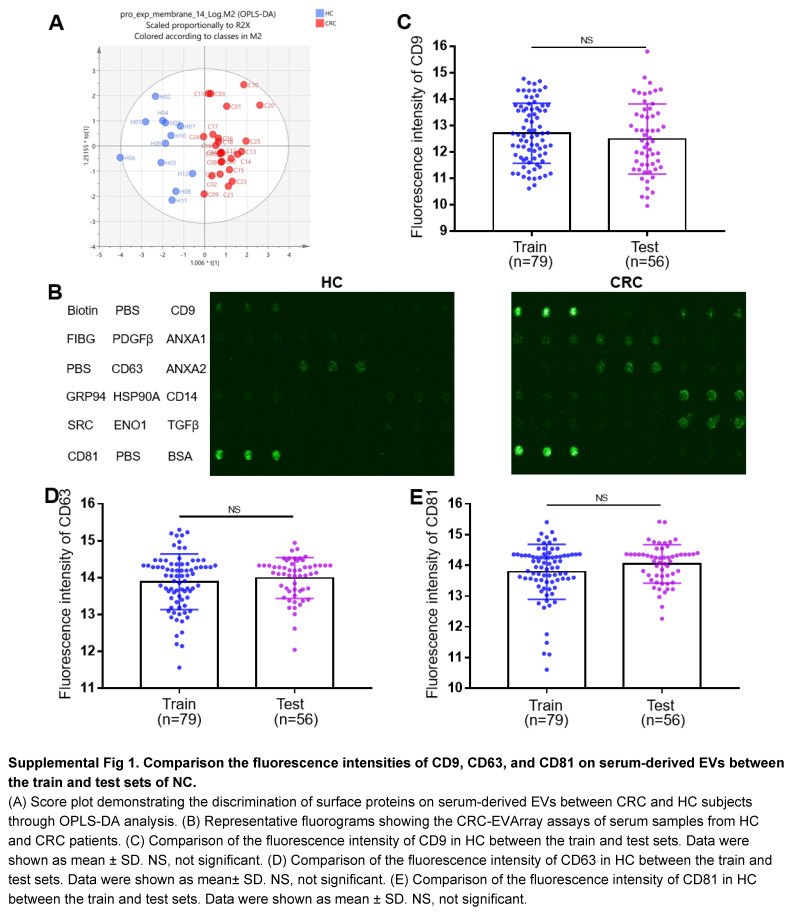


**Figure:**
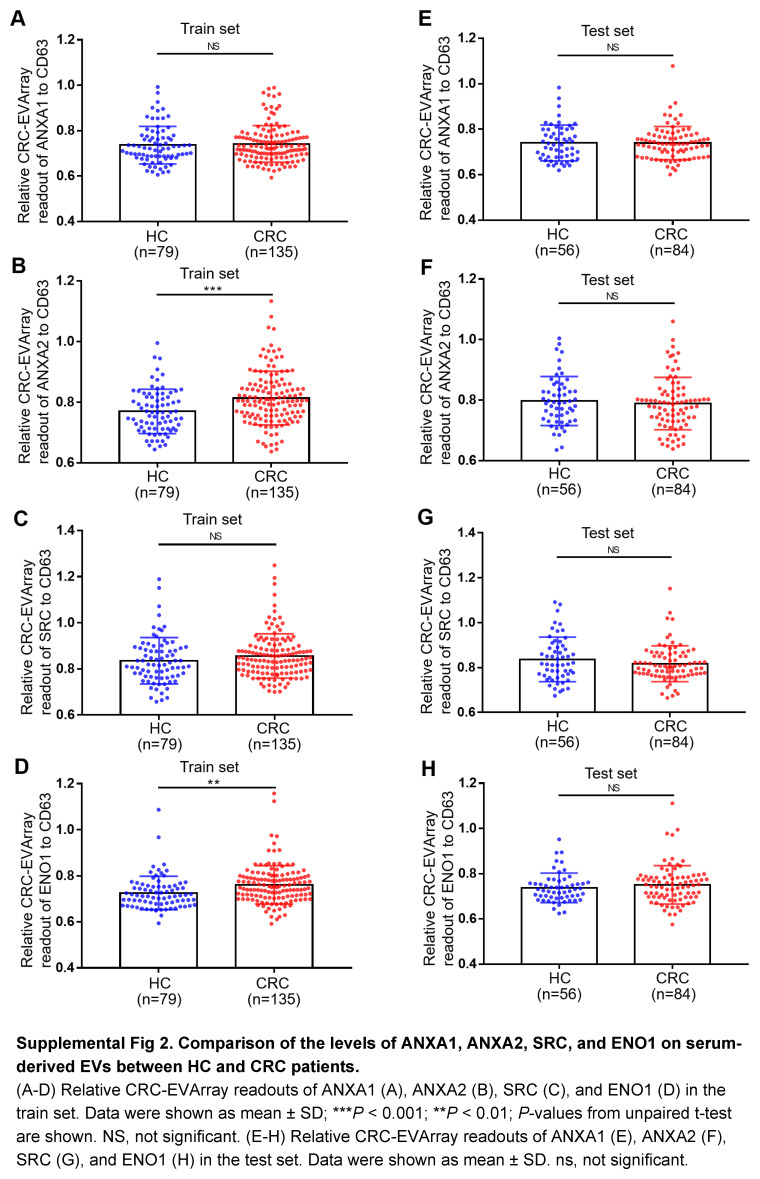


**Figure:**
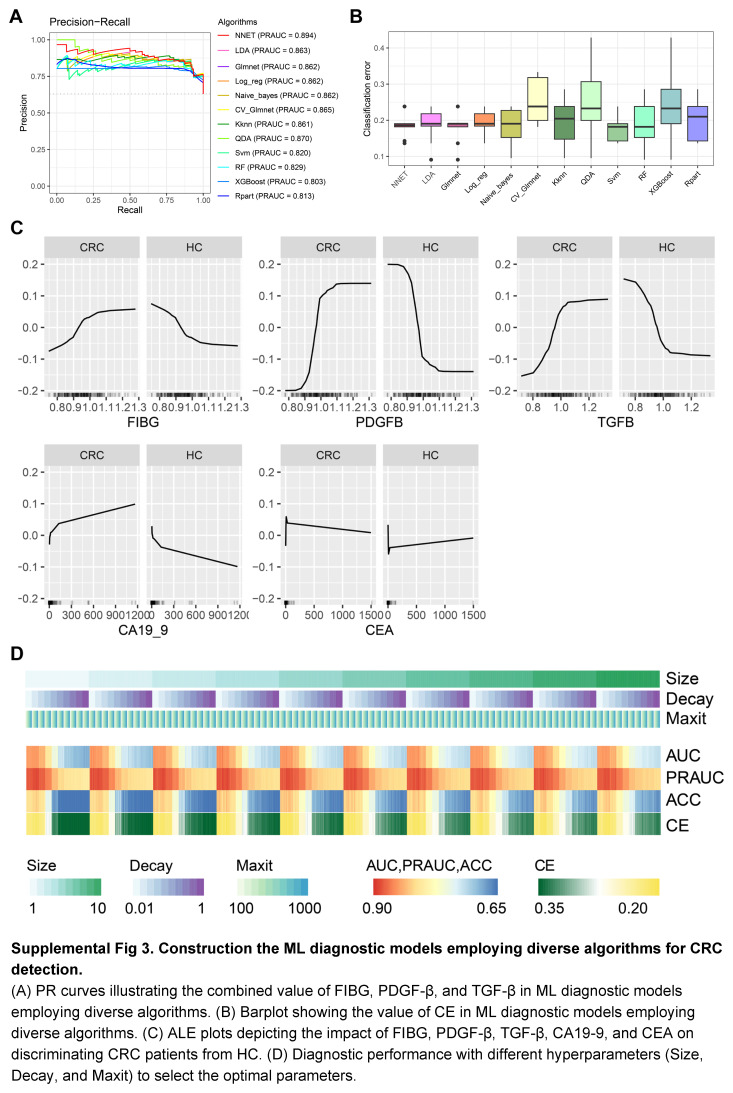


**Figure:**
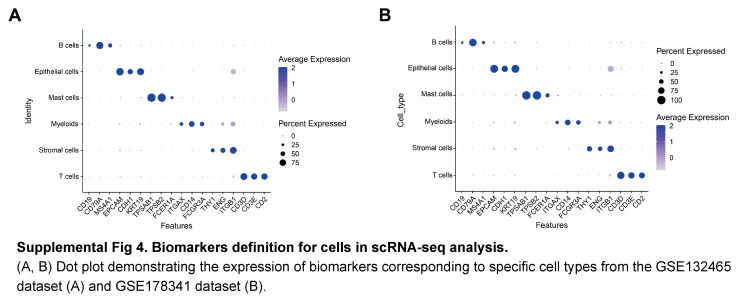


**Figure:**
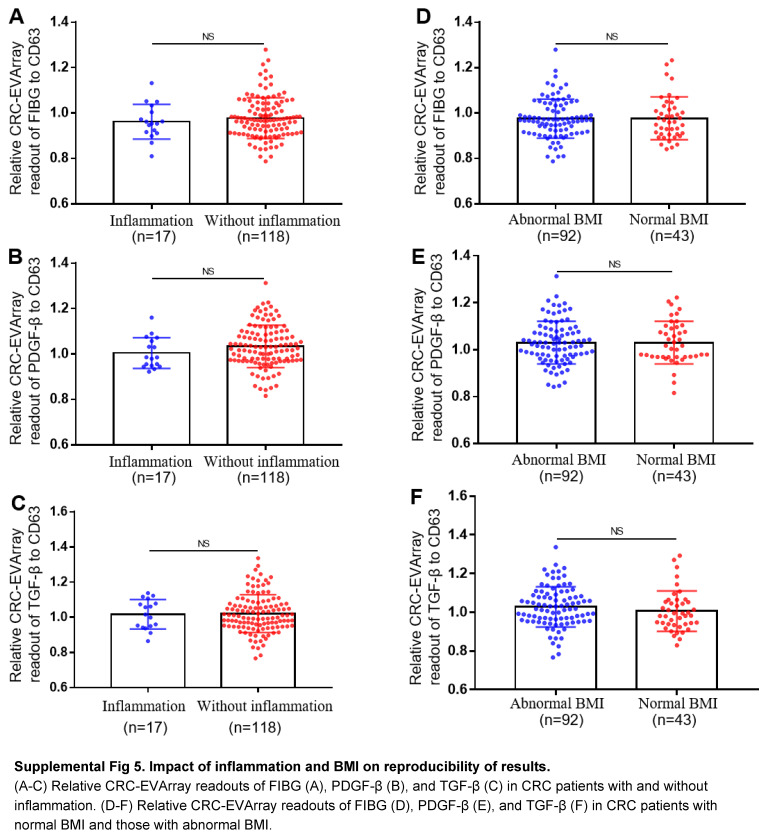


**Figure:**