# Supplementary material for: Identification and validation of the surface proteins FIBG, PDGF-β, and TGF-β on serum extracellular vesicles for non-invasive detection of colorectal cancer: experimental study
Source: Int J Surg. 2024 May 3;110(8):4672–87. doi: 10.1097/JS9.0000000000001533 (PMC11326011; doi:10.1097/JS9.0000000000001533)
Supplement: Supplementary file 1 [file js9-110-4672-s001.docx]

**Supplemental Table 1. Clinicopathologic Characteristics of Patients Included in the Study.**

| Characteristics | Discovery set  (n=37) | |  | Train set  (n=239) | | |  | Test set  (n=165) | | |
| --- | --- | --- | --- | --- | --- | --- | --- | --- | --- | --- |
|  | HC  (n=12) | CRC  (n=25) |  | HC  (n=79) | AA  (n=25) | CRC  (n=135) |  | HC  (n=56) | AA  (n=25) | CRC  (n=84) |
| Age, y, mean ± SD | 50.0±9.3 | 56.5±13.5 |  | 56.8±12.1 | 56.2±10.7 | 58.2±13.2 |  | 56.9±12.7 | 53.4±9.1 | 60.8±15.2 |
| Gender, n (%) |  |  |  |  |  |  |  |  |  |  |
| Male | 4 (33.3) | 13 (52.0) |  | 41 (51.9) | 16 (64.0) | 79 (58.5) |  | 30 (53.6) | 17 (68.0) | 47 (56.0) |
| Female | 8 (66.7) | 12 (48.0) |  | 38 (48.1) | 9 (36.0) | 56 (41.5) |  | 26 (46.4) | 8 (32.0) | 37 (44.0) |
| Clinical stage, n (%) |  |  |  |  |  |  |  |  |  |  |
| I | - | 3 (12.0) |  | - | - | 14 (10.4) |  | - | - | 11 (13.1) |
| II | - | 6 (24.0) |  | - | - | 46 (34.1) |  | - | - | 11 (13.1) |
| III | - | 8 (32.0) |  | - | - | 44 (32.6) |  | - | - | 48 (57.1) |
| IV | - | 4 (16.0) |  | - | - | 31 (23.0) |  | - | - | 14 (16.7) |
| Unknown | - | 4 (16.0) |  | - | - | 0 (0.0) |  | - | - | 0 (0.0) |
| CEA, ng/mL, n (%) |  |  |  |  |  |  |  |  |  |  |
| <5 | 12 (100.0) | 14 (56.0) |  | 77 (97.5) | 25 (100.0) | 96 (71.1) |  | 54 (96.4) | 25 (100.0) | 53 (63.1) |
| ≥5 | 0 (0.0) | 11 (44.0) |  | 2 (2.5) | 0 (0.0) | 39 (28.9) |  | 2 (3.6) | 0 (0.0) | 31 (36.9) |
| CA19-9, ng/mL, n (%) |  |  |  |  |  |  |  |  |  |  |
| <35 | 12 (100.0) | 18 (72.0) |  | 77 (97.5) | 25 (100.0) | 109 (80.7) |  | 56 (100.0) | 23 (92.0) | 65 (77.4) |
| ≥35 | 0 (0.0) | 7 (28.0) |  | 2 (2.5) | 0 (0.0) | 26 (19.3) |  | 0(0.0) | 2 (8.0) | 19 (22.6) |

**Supplemental Table 2. Analysis of the expression levels of FIBG on serum EVs in relation to the clinicopathological characteristics of CRC patients in the train and test sets.**

| Characteristics | Train set  (n=135) | | |  | Test set  (n=84) | | |
| --- | --- | --- | --- | --- | --- | --- | --- |
|  | No.of patients | serum EVs‐derived PF4 level (mean±SD) | *P* value |  | No.of patients | serum EVs‐derived PF4 level (mean±SD) | *P* value |
| Age |  |  |  |  |  |  |  |
| ≤70 | 111 | 0.979±0.090 | 0.329 |  | 60 | 0.965±0.096 | 0.330 |
| >70 | 24 | 0.960±0.078 |  |  | 24 | 0.987±0.081 |  |
| Gender, |  |  |  |  |  |  |  |
| Male | 79 | 0.973±0.087 | 0.698 |  | 47 | 0.979±0.098 | 0.398 |
| Female | 56 | 0.979±0.091 |  |  | 37 | 0.962±0.084 |  |
| Clinical stage |  |  |  |  |  |  |  |
| I-II | 60 | 0.943±0.069 | <0.001 |  | 22 | 0.918±0.060 | 0.001 |
| III-IV | 75 | 1.002±0.093 |  |  | 62 | 0.990±0.095 |  |
| T classification |  |  |  |  |  |  |  |
| T1-T2 | 30 | 0.955±0.087 | 0.003 |  | 23 | 0.930±0.069 | 0.010 |
| T3-T4 | 105 | 0.982±0.088 |  |  | 61 | 0.987±0.095 |  |
| N classification |  |  |  |  |  |  |  |
| N0 | 66 | 0.947±0.074 | <0.001 |  | 22 | 0.918±0.060 | 0.001 |
| N1-N3 | 69 | 1.003±0.093 |  |  | 62 | 0.990±0.095 |  |
| M classification |  |  |  |  |  |  |  |
| M0 | 104 | 0.967±0.080 | 0.032 |  | 70 | 0.966±0.092 | 0.199 |
| M1 | 31 | 1.005±0.107 |  |  | 14 | 1.000±0.093 |  |
| Differentiation |  |  |  |  |  |  |  |
| Poor | 17 | 0.967±0.121 | 0.608 |  | 15 | 0.970±0.081 | 0.680 |
| Moderate / Well | 96 | 0.979±0.083 |  |  | 28 | 0.981±0.090 |  |
| HER2 expression |  |  |  |  |  |  |  |
| Negative | 73 | 0.975±0.090 | 0.839 |  | 26 | 0.963±0.079 | 0.419 |
| Positive | 31 | 0.979±0.094 |  |  | 13 | 0.987±0.100 |  |
| pMMR / dMMR status |  |  |  |  |  |  |  |
| pMMR | 107 | 0.977±0.090 | 0.444 |  | 39 | 0.974±0.085 | 0.146 |
| dMMR | 9 | 0.953±0.061 |  |  | 4 | 0.910±0.032 |  |
| CEA (ng/mL) |  |  |  |  |  |  |  |
| <5 | 96 | 0.968±0.082 | 0.137 |  | 53 | 0.965±0.086 | 0.416 |
| ≥5 | 39 | 0.993±0.102 |  |  | 31 | 0.982±0.103 |  |
| CA19-9 (ng/mL) |  |  |  |  |  |  |  |
| <35 | 109 | 0.977±0.089 | 0.666 |  | 65 | 0.965±0.089 | 0.267 |
| ≥35 | 26 | 0.969±0.086 |  |  | 19 | 0.992±0.102 |  |

**Supplemental Table 3. Analysis of the expression levels of TGF-β on serum EVs in relation to the clinicopathological characteristics of CRC patients in the train and test sets.**

| Characteristics | Train set  (n=135) | | |  | Test set  (n=84) | | |
| --- | --- | --- | --- | --- | --- | --- | --- |
|  | No.of patients | serum EVs‐derived PF4 level (mean±SD) | *P* value |  | No.of patients | serum EVs‐derived PF4 level (mean±SD) | *P* value |
| Age |  |  |  |  |  |  |  |
| ≤70 | 111 | 1.032±0.089 | 0.535 |  | 60 | 1.029±0.097 | 0.135 |
| >70 | 24 | 1.019±0.100 |  |  | 24 | 0.996±0.074 |  |
| Gender, |  |  |  |  |  |  |  |
| Male | 79 | 1.026±0.110 | 0.440 |  | 47 | 1.009±0.110 | 0.690 |
| Female | 56 | 1.012±0.096 |  |  | 37 | 1.018±0.083 |  |
| Clinical stage |  |  |  |  |  |  |  |
| I-II | 60 | 0.989±0.092 | 0.001 |  | 22 | 0.962±0.056 | 0.004 |
| III-IV | 75 | 1.046±0.107 |  |  | 62 | 1.031±0.105 |  |
| T classification |  |  |  |  |  |  |  |
| T1-T2 | 30 | 1.010±0.101 | 0.532 |  | 23 | 0.964±0.081 | 0.005 |
| T3-T4 | 105 | 1.024±0.106 |  |  | 61 | 1.031±0.099 |  |
| N classification |  |  |  |  |  |  |  |
| N0 | 66 | 1.000±0.108 | 0.026 |  | 22 | 0.962±0.056 | 0.004 |
| N1-N3 | 69 | 1.0406±0.098 |  |  | 62 | 1.031±0.105 |  |
| M classification |  |  |  |  |  |  |  |
| M0 | 104 | 1.004±0.102 | 0.001 |  | 70 | 1.000±0.091 | 0.007 |
| M1 | 31 | 1.076±0.095 |  |  | 14 | 1.077±0.116 |  |
| Differentiation |  |  |  |  |  |  |  |
| Poor | 17 | 1.002±0.114 | 0.377 |  | 15 | 1.030±0.110 | 0.779 |
| Moderate / Well | 96 | 1.028±0.108 |  |  | 28 | 1.040±0.112 |  |
| HER2 expression |  |  |  |  |  |  |  |
| Negative | 73 | 1.011±0.113 | 0.280 |  | 26 | 1.032±0.108 | 0.590 |
| Positive | 31 | 1.036±0.100 |  |  | 13 | 1.052±0.106 |  |
| pMMR / dMMR status |  |  |  |  |  |  |  |
| pMMR | 107 | 1.014±0.108 | 0.050 |  | 39 | 1.046±0.110 | 0.097 |
| dMMR | 9 | 1.087±0.078 |  |  | 4 | 0.951±0.062 |  |
| CEA (ng/mL) |  |  |  |  |  |  |  |
| <5 | 96 | 1.016±0.106 | 0.433 |  | 53 | 0.990±0.089 | 0.004 |
| ≥5 | 39 | 1.032±0.101 |  |  | 31 | 1.052±0.103 |  |
| CA19-9 (ng/mL) |  |  |  |  |  |  |  |
| <35 | 109 | 1.013±0.106 | 0.085 |  | 65 | 1.001±0.090 | 0.051 |
| ≥35 | 26 | 1.052±0.090 |  |  | 19 | 1.052±0.119 |  |

**Supplemental Table 4. Analysis of the expression levels of PDGF-β on serum EVs in relation to the clinicopathological characteristics of CRC patients in the train and test sets.**

| Characteristics | Train set  (n=135) | | |  | Test set  (n=84) | | |
| --- | --- | --- | --- | --- | --- | --- | --- |
|  | No.of patients | serum EVs‐derived PF4 level (mean±SD) | *P* value |  | No.of patients | serum EVs‐derived PF4 level (mean±SD) | *P* value |
| Age |  |  |  |  |  |  |  |
| ≤70 | 111 | 1.026±0.100 | 0.132 |  | 60 | 1.007±0.106 | 0.420 |
| >70 | 24 | 0.990±0.116 |  |  | 24 | 1.027±0.080 |  |
| Gender, |  |  |  |  |  |  |  |
| Male | 79 | 1.030±0.094 | 0.982 |  | 47 | 1.018±0.100 | 0.892 |
| Female | 56 | 1.030±0.086 |  |  | 37 | 1.021±0.081 |  |
| Clinical stage |  |  |  |  |  |  |  |
| I-II | 60 | 1.015±0.085 | 0.083 |  | 22 | 0.991±0.062 | 0.088 |
| III-IV | 75 | 1.042±0.094 |  |  | 62 | 1.029±0.098 |  |
| T classification |  |  |  |  |  |  |  |
| T1-T2 | 30 | 1.014±0.093 | 0.271 |  | 23 | 1.012±0.080 | 0.657 |
| T3-T4 | 105 | 1.034±0.090 |  |  | 61 | 1.022±0.096 |  |
| N classification |  |  |  |  |  |  |  |
| N0 | 66 | 1.020±0.086 | 0.227 |  | 22 | 0.991±0.062 | 0.088 |
| N1-N3 | 69 | 1.039±0.095 |  |  | 62 | 1.029±0.098 |  |
| M classification |  |  |  |  |  |  |  |
| M0 | 104 | 1.026±0.092 | 0.368 |  | 70 | 1.016±0.089 | 0.433 |
| M1 | 31 | 1.043±0.088 |  |  | 14 | 1.037±0.105 |  |
| Differentiation |  |  |  |  |  |  |  |
| Poor | 17 | 0.990±0.083 | 0.040 |  | 15 | 1.012±0.090 | 0.732 |
| Moderate / Well | 96 | 1.040±0.094 |  |  | 28 | 1.023±0.100 |  |
| HER2 expression |  |  |  |  |  |  |  |
| Negative | 73 | 1.032±0.098 | 0.546 |  | 26 | 1.014±0.091 | 0.400 |
| Positive | 31 | 1.020±0.083 |  |  | 13 | 1.041±0.107 |  |
| pMMR / dMMR status |  |  |  |  |  |  |  |
| pMMR | 107 | 1.027±0.093 | 0.935 |  | 39 | 1.025±0.093 | 0.435 |
| dMMR | 9 | 1.029±0.088 |  |  | 4 | 0.987±0.079 |  |
| CEA (ng/mL) |  |  |  |  |  |  |  |
| <5 | 96 | 1.024±0.088 | 0.263 |  | 53 | 1.009±0.089 | 0.182 |
| ≥5 | 39 | 1.044±0.096 |  |  | 31 | 1.037±0.095 |  |
| CA19-9 (ng/mL) |  |  |  |  |  |  |  |
| <35 | 109 | 1.031±0.091 | 0.700 |  | 65 | 1.010±0.091 | 0.097 |
| ≥35 | 26 | 1.024±0.089 |  |  | 19 | 1.050±0.089 |  |

**Supplemental Table 5. Performance of different ML models.**

| ML Algorithms | AUC | PRAUC | Classification error | Sensitivity | Specificity | Precision | Recall | Accuracy | F1 score |
| --- | --- | --- | --- | --- | --- | --- | --- | --- | --- |
| NNET | 0.863 | 0.894 | 0.187 | 0.904 | 0.673 | 0.821 | 0.904 | 0.813 | 0.860 |
| LDA | 0.854 | 0.863 | 0.187 | 0.920 | 0.651 | 0.812 | 0.920 | 0.813 | 0.863 |
| CV_Glmnet | 0.852 | 0.865 | 0.253 | 0.947 | 0.432 | 0.734 | 0.947 | 0.747 | 0.827 |
| Glmnet | 0.852 | 0.862 | 0.183 | 0.925 | 0.651 | 0.813 | 0.925 | 0.817 | 0.865 |
| Log_reg | 0.852 | 0.862 | 0.197 | 0.906 | 0.651 | 0.811 | 0.906 | 0.803 | 0.856 |
| Naive_bayes | 0.851 | 0.862 | 0.187 | 0.841 | 0.767 | 0.857 | 0.841 | 0.813 | 0.849 |
| Kknn | 0.841 | 0.862 | 0.192 | 0.886 | 0.687 | 0.823 | 0.886 | 0.808 | 0.854 |
| QDA | 0.821 | 0.871 | 0.247 | 0.798 | 0.693 | 0.814 | 0.798 | 0.753 | 0.806 |
| Svm | 0.812 | 0.820 | 0.178 | 0.926 | 0.662 | 0.818 | 0.926 | 0.822 | 0.869 |
| RF | 0.797 | 0.829 | 0.187 | 0.907 | 0.659 | 0.813 | 0.907 | 0.813 | 0.857 |
| XGBoost | 0.789 | 0.803 | 0.244 | 0.853 | 0.603 | 0.775 | 0.853 | 0.756 | 0.812 |
| Rpart | 0.784 | 0.813 | 0.201 | 0.932 | 0.589 | 0.787 | 0.932 | 0.799 | 0.853 |

**Supplemental Table 6. Diagnostic performance of NNET models based on different combinations.**

| Combinations | AUC | PRAUC | Classification error | Sensitivity | Specificity | Precision | Recall | Accuracy | F1 score |
| --- | --- | --- | --- | --- | --- | --- | --- | --- | --- |
| TGFB | 0.796 | 0.847 | 0.266 | 0.842 | 0.558 | 0.748 | 0.842 | 0.734 | 0.792 |
| PDGFB-CEA | 0.850 | 0.881 | 0.192 | 0.884 | 0.687 | 0.825 | 0.884 | 0.808 | 0.853 |
| PDGFB-TGFB-CEA | 0.868 | 0.890 | 0.181 | 0.898 | 0.722 | 0.827 | 0.898 | 0.819 | 0.861 |
| FIBG-PDGFB-TGFB-CEA | 0.870 | 0.890 | 0.186 | 0.888 | 0.717 | 0.826 | 0.888 | 0.814 | 0.856 |
| FIBG-PDGFB-TGFB-CA19_9-CEA | 0.804 | 0.851 | 0.266 | 0.886 | 0.517 | 0.764 | 0.886 | 0.734 | 0.821 |

**Supplemental Table 7. Diagnostic performances of the train and test sets through the CRC-EVArray diagnostic model.**

| Models | AUC | PRAUC | Classification error | Sensitivity | Specificity | Precision | Recall | Accuracy | F1 score |
| --- | --- | --- | --- | --- | --- | --- | --- | --- | --- |
| The EVs-surface proteins-related model | 0.882 | 0.917 | 0.187 | 0.888 | 0.731 | 0.845 | 0.888 | 0.813 | 0.866 |
| Test set (CRC vs HC) | 0.937 | 0.963 | 0.171 | 0.929 | 0.679 | 0.813 | 0.929 | 0.829 | 0.867 |
| Test set (Stage Ⅰ-Ⅱ CRC vs HC) | 0.821 | 0.682 | 0.269 | 0.773 | 0.714 | 0.515 | 0.773 | 0.731 | 0.618 |
| Train set (AA vs HC) | 0.822 | 0.637 | 0.250 | 0.760 | 0.747 | 0.487 | 0.760 | 0.750 | 0.594 |
| Test set (AA vs HC) | 0.767 | 0.543 | 0.296 | 0.680 | 0.714 | 0.515 | 0.680 | 0.704 | 0.586 |
